# A Lesson into Decolonized Psychiatric and Psychoanalytic Practice: Demystifying the Works of Girindrasekhar Bose

**DOI:** 10.1192/j.eurpsy.2023.2072

**Published:** 2023-07-19

**Authors:** V. Gairola

**Affiliations:** Department of Liberal Arts, Indian Institute of Technology, Hyderabad, Noida, India

## Abstract

**Introduction:**

The endeavor of practitioners of psychiatry and psychoanalysis has been to break the disciplinary silos and to re-think what practice is all about. There was a person who pioneered this aim of intradisciplinary and interdisciplinary synthesis. His name was Girindrasekhar Bose. He was a psychiatrist, psychologist, and psychoanalyst who challenged the Freudian universalism of the Oedipus complex.

**Objectives:**

We have forgotten that the texts emanating from the land of India were profoundly psychological and can inform psychiatric/psychoanalytic practice. Bose’s exchange of letters with Sigmund Freud is known by many but read by only a few. He gave us a path to think about the psychiatric and psychoanalytic practice *from* India. The paper looks at his exchange of letters with Freud and his works on the Bhagavad Gita, Puranas, Yoga Sutras, and the Vedas, which he claimed, were immensely beneficial for psychiatric/psychoanalytic training and can help us to work through the way we perceive medical practice.

**Methods:**

This research used primary sources like books and articles to re-think the works of Girindrasekhar Bose in terms of today’s medical practice.

**Results:**

Can we imagine writing in our native language and being critically acclaimed for our work internationally? His writings in Bengali suggest that he was more decolonized in the 1920s than the Indian scholars of today. Keeping in mind the existential ground of the patient, a decolonized understanding of symptoms can revolutionize medical care.

**Conclusions:**

Our aim in today’s times is to inform our practice where we could possibly reach an intradisciplinary and interdisciplinary unity. A re-discovery of Bose’s work can help us in this earnest endeavor.

**Disclosure of Interest:**

None Declared

